# Oleuropein Stimulates Migration of Human Trophoblast Cells and Expression of Invasion-Associated Markers

**DOI:** 10.3390/ijms25010500

**Published:** 2023-12-29

**Authors:** Andrea Pirković, Milica Jovanović Krivokuća, Aleksandra Vilotić, Mirjana Nacka-Aleksić, Žanka Bojić-Trbojević, Dragana Dekanski

**Affiliations:** Institute for the Application of Nuclear Energy, Department for Biology of Reproduction, University of Belgrade, Banatska 31b, 11080 Belgrade, Serbia; andrea.pirkovic@inep.co.rs (A.P.); milicaj@inep.co.rs (M.J.K.); aleksandrav@inep.co.rs (A.V.); mnacka@inep.co.rs (M.N.-A.); zana@inep.co.rs (Ž.B.-T.)

**Keywords:** oleuropein, pregnancy, trophoblast function, cell migration, invasion

## Abstract

Successful pregnancy establishment requires highly synchronized cross talk between the invasive trophoblast cells and the receptive maternal endometrium. Any disturbances in this tightly regulated process may lead to pregnancy complications. Local factors such as nutrients, hormones, cytokines and reactive oxygen species modulate the invasion of extravillous trophoblasts through critical signaling cascades. Epidemiological studies strongly indicate that a Mediterranean diet can significantly impact molecular pathways during placentation. Therefore, the aim of the current study was to examine whether oleuropein (OLE), one of the main compounds of the Mediterranean diet, may influence trophoblast cell adhesion and migration, as well as the expression of invasion-associated molecular markers and inflammatory pathways fostering these processes. HTR-8/SVneo cells were incubated with OLE at selected concentrations of 10 and 100 µM for 24 h. Results showed that OLE did not affect trophoblast cell viability, proliferation and adhesion after 24 h in in vitro treatment. The mRNA expression of integrin subunits α1, α5 and β1, as well as matrix-degrading enzymes MMP-2 and -9, was significantly increased after treatment with 10 µM OLE. Furthermore, OLE at a concentration of 10 µM significantly increased the protein expression of integrin subunits α1 and β1. Also, OLE inhibited the activation of JNK and reduced the protein expression of COX-2. Finally, a lower concentration of OLE 10 µM significantly stimulated migration of HTR-8/SVneo cells. In conclusion, the obtained results demonstrate the effects of OLE on the function of trophoblast cells by promoting cell migration and stimulating the expression of invasion markers. As suggested from results, these effects may be mediated via inhibition of the JNK signaling pathway.

## 1. Introduction

The placenta is the temporary organ developed during pregnancy and is of vital importance for transporting oxygen and nutrients between the mother and fetus [1]. The crucial functions of the placenta in the early first trimester of pregnancy are performed by trophoblast cells which form the feto-maternal interface, a functional connection between the mother and the fetus. As the specific type of trophoblast cells, extravillous trophoblast cells (EVTs) are inherently invasive, and their role is to invade the maternal endometrium, spiral arteries and other luminal structures in the uterus, and adjust them for the further progress of pregnancy [2,3]. The outcome of this process is an adequately attached placenta and modified maternal spiral arteries that enable sufficient supply of oxygen, nutrients and other factors to the developing fetus. These processes require a highly controlled and synchronized interaction of the maternal and fetal side, and are tightly related to the impact of nutrients, hormones and environmental factors. Considering the central role of trophoblast cells in placental development, examination of key regulatory factors and signaling cascades governing placentation are being examined in trophoblast cell lines as appropriate cellular model systems for detailed molecular investigations [2].

In normal early pregnancy, local oxidative stress and inflammatory response are increased, resulting in increased production of reactive oxygen species (ROS) and cytokines at the feto–maternal interface. Signaling networks of these molecules play an important role in regulation of trophoblast adhesion, invasion, and remodeling of the maternal spiral arteries. In the earliest stages of pregnancy, a signaling network of ROS and cytokines can either stimulate or limit the extent of trophoblast invasion and plays an important role in proper placentation. However, uncontrolled and excessive oxidative stress and inflammation at feto–maternal interface could lead to a detrimental impact on vascularization and placentation and cause development of placental pathologies and pregnancy disorders [4]. The connection between oxidative stress and inflammation is proposed as the main mechanism promoting placental dysfunction in pregnancy [5]. The use of antioxidants and modified diet represents a possible approach in the prevention of such disorders [6].

Regarding pregnancy, nutrition may have an important role in achieving optimal metabolic balance for both the mother and the fetus [7]. Epidemiological studies strongly indicate that nutrition can significantly impact several pathways during placentation and the course of pregnancy [8]. Animal models have shown that maternal undernutrition and exposure to adverse external factors can negatively affect proliferation and expansion of cells in the placenta leading to intrauterine growth restriction [9]. In women with obesity and infertility, a preconception nutritional-lifestyle intervention initiated prior to fertility treatments showed success in improving reproductive outcomes and demonstrated positive effects on markers of adiposity and pro-inflammatory states [10,11]. In this context, the Mediterranean diet (MD) is recognized for its benefits. It is known that strong adherence to a MD is associated with a lower incidence of pregnancy complications [12,13]. However, the direct impact of diet and lifestyle on placental cell function is largely unknown. MD is low in saturated fats and enriched with bioactive food components like polyphenols, vitamins and minerals, essentially derived from extra virgin olive oil. The beneficial effects of MD are mainly ascribed to the promotion of the antioxidant function and attenuation of oxidative stress in cells. Some of these bioactive components of MD have shown the potential to regulate cellular signaling pathways, modulate gene expression, affect transcription factors and alter the microRNAs’ (miRNAs) profile [14]. Major phenolic compounds found within MD, such as hydroxytyrosol, oleuropein (OLE) and resveratrol, have shown benefits in atherosclerosis and polycystic ovary syndrome, by inhibiting the expression of cytokines, chemokines and adhesion molecules induced by inflammatory stimuli [15,16,17]. Thus, the use of antioxidants such as the bioactive polyphenol OLE could be a possible approach in the prevention of inflammation- and oxidative stress-related pregnancy disorders as well [18].

OLE is known for its remarkable antioxidant and anti-inflammatory activity and its use is associated with numerous health benefits [19,20,21,22]. In in vivo study, OLE treatment suppressed the growth in ectopic lesions in mice with endometriosis and remarkably increased pregnancy rate (100%) compared to vehicle-treated mice (70%) [23]. In other study performed on mice with gestational diabetes mellitus (GDM), OLE efficiently reduced blood glucose, insulin and alleviated oxidative stress and inflammation [24]. However, its role in human pregnancy is not examined. As we have shown in our recent research, in the first step towards examining the possible effects of OLE in pregnancy, OLE exhibited significant cytoprotective effects in trophoblast HTR-8/SVneo cells in the model of oxidative stress caused by H_2_O_2_ [25]. Namely, it improved the antioxidant status of cells, prevented protein oxidation and reduced lipid peroxidation. Also, the anti-inflammatory effect of OLE was demonstrated through inhibition of inducible nitric oxide synthase (iNOS) expression and reduction in mRNA expression of pro-inflammatory cytokines TNF-α and IL-6 in trophoblast cells exposed to H_2_O_2_. To further elucidate the impact of OLE in pregnancy, it is necessary to examine its influence on the molecular pathways involved in the regulation of key functional processes, such as trophoblast invasion. As previously mentioned, local inflammation at the feto–maternal interface is a critical regulator of EVT morphology and function. Stimulation of pro-inflammatory mediators such as iNOS and cyclooxygenase-2 (COX-2), which may activate c-Jun NH2-terminal kinase (JNK) signaling, is enhanced in pathological placentas [26]. Excess inflammation in the maternal decidua followed by immune disturbance is known to reduce trophoblast invasion causing shallow placentation [27]. Thus, the aim of the current study was to examine whether OLE may influence trophoblast adhesion, migration and invasion by modulating the expression and activity of relevant molecular markers and inflammatory pathways fostering these processes. These findings may contribute to the formulation of functional food-based guidelines for improving metabolic and reproductive outcomes in pregnancy. 

## 2. Results

### 2.1. Viability of Oleuropein Treated HTR-8/SVneo Cells Determined by Crystal Violet Assay

**Figure 1:**
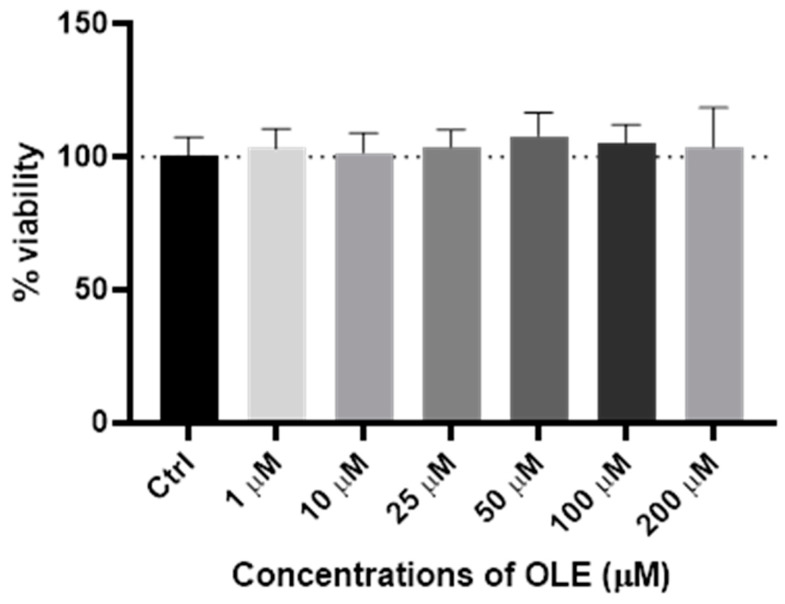
Viability of HTR-8/SVneo cells following 24 h incubation period with different concentrations of oleuropein (OLE) compared to untreated control (Ctrl), determined by crystal violet assay.

Results presented on Figure 1 showed no significant change in the number of adherent cells after the 24 h exposure to OLE in the range of concentrations from 1 to 200 µM, indicating lack of cytotoxic effects of OLE to HTR-8/SVneo cells. Even at the highest concentration of 200 µM, there was no significant change in the number of adherent cells compared to unexposed control. Based on the results of cell viability presented in Figure 1, the two concentrations of OLE were chosen for further tests: 10 µM of OLE was chosen for investigation of low-dose effects while concentration of 100 µM OLE was chosen for evaluation of high-dose OLE effects on HTR-8/SVneo cells. 

### 2.2. Effects of Oleuropein on the Proliferation of HTR-8/SVneo Cells

**Figure 2:**
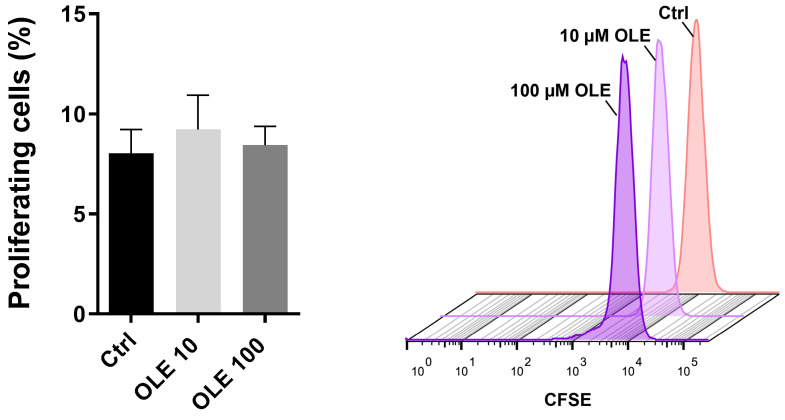
Effects of 10 µM and 100 µM oleuropein (OLE) on proliferation of HTR-8/SVneo cells. The bar graph indicates the frequency of CFSE+ (proliferating cells) among unstimulated cells (Ctrl) and cells stimulated with either 10 µM or 100 µM OLE. The representative flow cytometry histograms show CFSE dilution in HTR-8/SVneo cells.

The results (Figure 2) show the direct effect of OLE on trophoblast cells proliferation. A fluorescent dye, CFSE, was used to monitor cell proliferation for 48 h and calculate the frequency of proliferating cells. As cells undergo division, the CFSE fluorescence intensity is being divided evenly between the two daughter cells, so it is an indirect measure of the number of cell divisions. By comparing the frequencies of proliferating cells among the cells treated with two different concentrations of OLE (10 and 100 μM) and untreated controls (Figure 2 left panel), it was found that OLE does not change the proliferation of HTR-8/SVneo cells in any of the used concentrations. The histogram in Figure 2 (right panel) shows the number of cells as a function of CFSE fluorescence intensity. It can be observed that there was no difference between the numbers of proliferating cells in OLE treatments 10 and 100 μM and untreated control. 

### 2.3. Effect of Oleuropein on the Substrate-Dependent Adhesion of HTR-8/SVneo Cells to Plastic, Collagen and Matrigel

**Figure 3:**
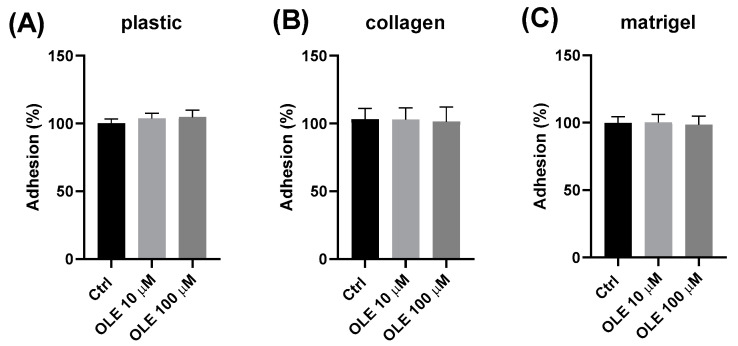
Effect of oleuropein (OLE) on the substrate-dependent adhesion of HTR-8/SVneo cells to: plastic (**A**), collagen (**B**) and Matrigel (**C**).

The results in Figure 3 show the adhesion of HTR-8/SVneo cells on a surface coated with either Matrigel or collagen type I as well as on an uncoated plastic well surface, following the pre-incubation treatment with OLE for 24 h. The obtained results show that the pre-treatment of cells with OLE had no significant effect on the adhesion of HTR-8/SVneo cells compared to the control ones on any of the tested substrates. There was a slight increase in cell adhesion on plastic of the cells treated with OLE; however, the observed difference was not statistically significant.

### 2.4. Effect of Oleuropein on the Relative mRNA Expression of Matrix Metalloproteinases 2 and 9 and Integrin Subunits α1, α5 and β1 in HTR-8/SVneo Cells

**Figure 4:**
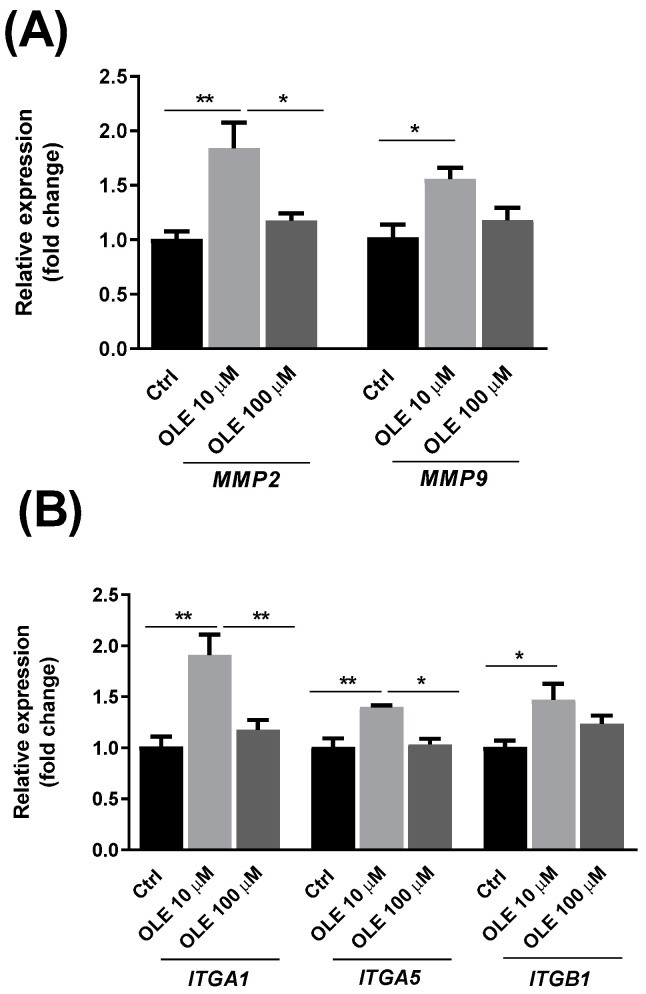
Effect of oleuropein (OLE) on the relative mRNA expression of matrix metalloproteinases 2 and 9 (*MMP2*, *MMP9*) (**A**) and integrin subunits α1, α5 and β1 (*ITGA1*, *ITGA5*, *ITGB1*) (**B**) in HTR-8/SVneo cells. * *p* < 0.05; ** *p* < 0.01.

After it was determined that OLE does not affect the adhesion process of HTR-8/SVneo cells, we further wanted to examine whether the expression of molecular factors mediating the process of trophoblast invasion is affected by the treatment with OLE. Figure 4 represents the effect of OLE treatment on the expression of proteolytic and adhesion molecules that are important for the degradation of the extracellular matrix (ECM) and the invasion of trophoblast cells. The mRNA expression of matrix metalloproteinases *MMP2*, *MMP9* and integrin subunits α1, α5 and β1 (*ITGA1*, *ITGA5*, *ITGB1*) was examined in HTR-8/SVneo cells 24 h after OLE treatment, by using the quantitative real-time PCR (qPCR) method. OLE at a lower concentration of 10 µM induced a significant increase in the mRNA expression of all examined integrin subunits (*ITGA1*, *ITGA5* and *ITGB1*). On the other hand, OLE at a concentration of 100 µM did not lead to a significant change in the mRNA expression of the mentioned integrin subunits. The lower concentration of OLE (10 µM) also led to the increased mRNA expression of *MMP2* and *MMP9* compared to the expression level in untreated cells, while no significant change was observed after the treatment with 100 µM OLE.

### 2.5. Effect of Oleuropein on the Expression of Integrin Subunits α1 and β1 in HTR-8/SVneo Cells at the Protein Level

**Figure 5:**
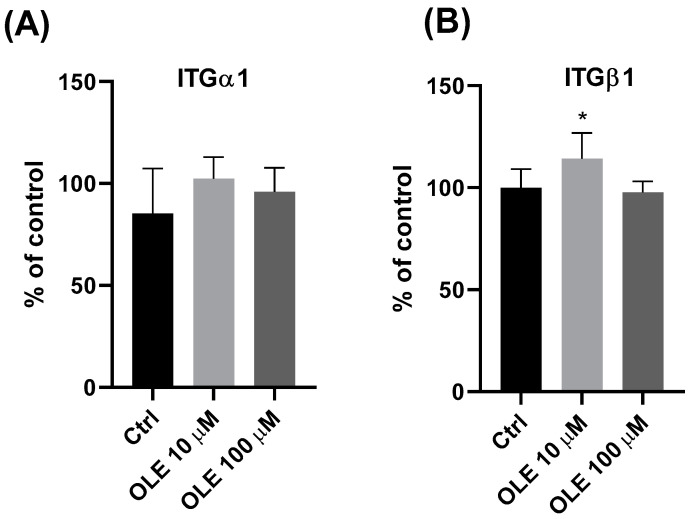
Effect of oleuropein (OLE) on the expression of integrin subunits α1 (ITGα1) (**A**) and β1 (ITGβ1) (**B**) in HTR-8/SVneo cells at the protein level, using the CELISA method. * *p* < 0.05.

Examining the effect of OLE on integrin subunits α1 and β1 at the protein level, using the CELISA (cell-based ELISA) method, it was shown that OLE at a concentration of 10 µM significantly increased the integrin β1 subunit expression (Figure 5B). A similar trend was observed for the expression of α1 integrin subunit (Figure 5A), where 10 µM OLE increased expression compared to the control; however, the changes were not statistically significant due to large deviations in the control. A concentration of OLE at 100 µM showed no significant effect on the expression of integrin subunit α1 and β1 at the protein level. Since CELISA requires live, adherent cells for measurement, and is used for protein quantification in whole cells, it can be concluded that OLE 10 μM can significantly stimulate the expression of integrin subunits α1 and β1 on protein level in live cultured cells. 

### 2.6. Effect of Oleuropein on the Expression of Cyclooxygenase 2 in HTR-8/SVneo Cells at the Protein Level

**Figure 6:**
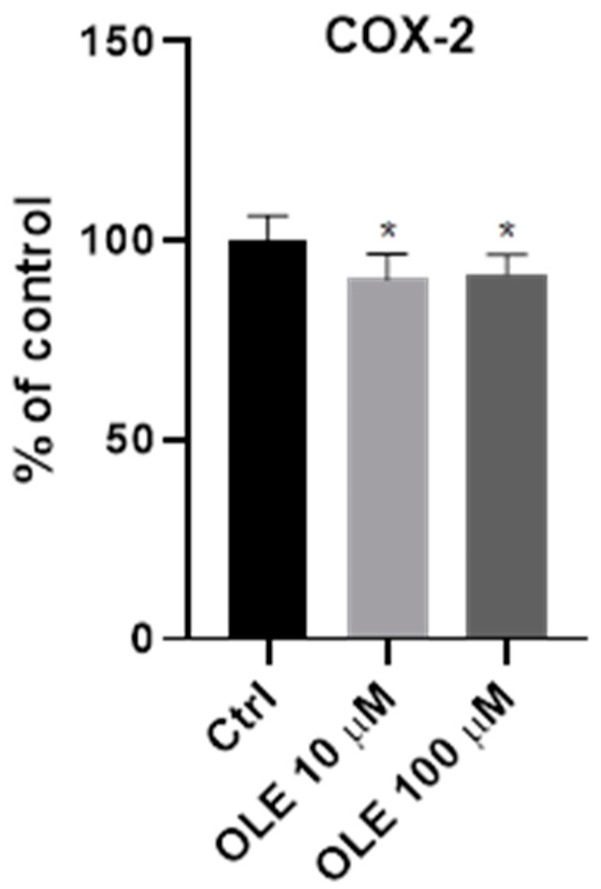
Effect of oleuropein (OLE) on the expression of cyclooxygenase 2 (COX-2) in HTR-8/SVneo cells at the protein level, using the CELISA method. * *p* < 0.05.

Since it is known that COX-2 plays an important role in the regulation of human trophoblast cell invasion [28], the effects of OLE on the protein expression of COX-2 were examined after 24 h incubation in HTR-8/SVneo cells. Here, by using CELISA, an immunocytochemistry method used to quantify target protein, COX-2 was evaluated at the protein level. The results presented in Figure 6 show that there is a moderate yet significant inhibition of COX-2 expression in trophoblast cells treated with OLE. Furthermore, there was a significant difference at both treatments with 10 and 100 µM OLE compared to untreated control, confirming the inhibitory effect of OLE on COX-2 expression at protein level. 

### 2.7. Effect of Oleuropein on JNK Signaling Pathway in HTR-8/SVneo Cells

**Figure 7:**
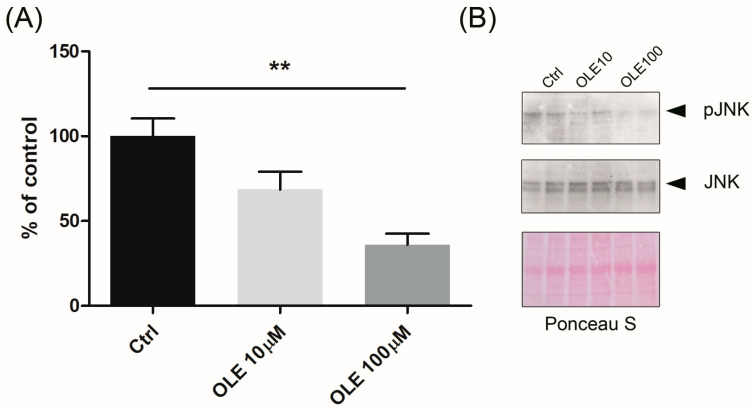
Effect of oleuropein (OLE) on JNK signaling pathway in HTR-8/SVneo cells. (**A**) Ratio of pJNK/JNK was calculated following densitometry analysis of blots and results are presented as percentage of the obtained values for the control cells. Representative blots are presented in (**B**). ** *p* < 0.01.

To further examine the molecular mechanism behind the effect of OLE in trophoblast cells, we also determined its role on c-Jun N-terminal kinase (JNK) activation by Western blot analysis. Cells were incubated in absence or presence of two different concentrations of OLE (10 µM and 100 µM) for 24 h and the appearance of phosphorylated JNK (pJNK) was monitored. OLE treatment was shown to inhibit JNK activation in a concentration dependent manner (Figure 7). The higher concentration of 100 µM OLE significantly reduced the expression of pJNK.

### 2.8. Effect of Oleuropein on HTR-8/SVneo Cell Migration

**Figure 8:**
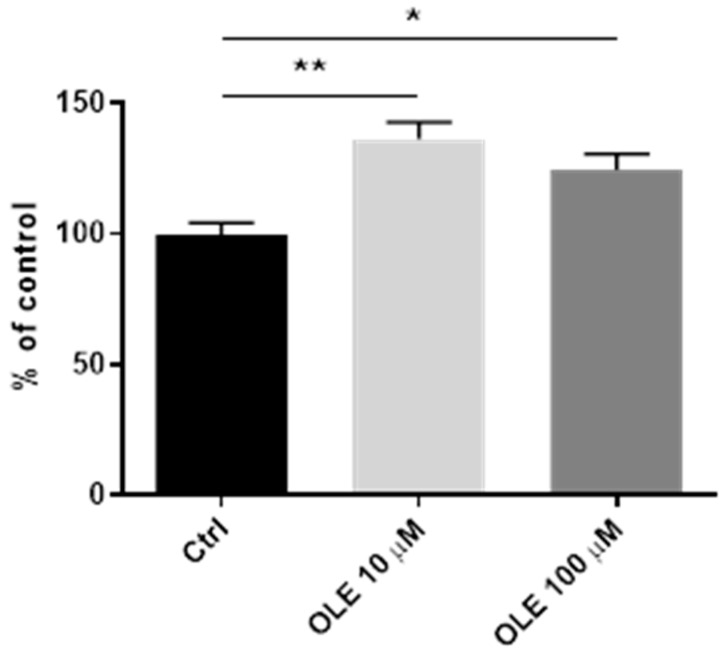
Effect of oleuropein (OLE) on HTR-8/SVneo cell migration after 24 h of culture, determined by in vitro scratch-wound migration assay. The trophoblast migration was calculated based on the distances crossed by the cells which were measured using an electronic grid (0.1 × 0.1 mm^2^). Data (from three experiments, and two areas measured per well) are expressed as the percentage of the untreated control. * *p* < 0.05; ** for *p* < 0.01.

The potential of OLE to influence cell migration at effective concentrations of 10 and 100 µM in HTR-8/SVneo cells after 24 h of culture is shown on Figure 8. The results show a concentration-dependent effect, with both concentrations of OLE having significant stimulatory effects on HTR-8/SVneo cell migration (136% for OLE 10 µM and 124% for OLE 100 µM, of the control values) after 24 h of culture. When compared, the stimulatory effects of 10 µM OLE concentration on cell migration were more pronounced than 100 µM OLE.

## 3. Discussion

Trophoblast invasion is a crucial process in the first trimester of gestation. In uncomplicated, normal pregnancies, EVTs invade the decidua and maternal spiral arteries, and subsequently remodel them to allow maternal blood flow towards the intervillous space of the placenta [29]. The invasive trophoblast cells have highly specialized behavior, which includes attachment to the proteins of the ECM, secretion of proteinases capable of degrading the ECM, and migration through the degraded matrix [30]. Trophoblast invasiveness is tightly controlled by local factors, both trophoblast-derived as well as maternal factors in a time- and spatially dependent manner [31]. Pregnancy disorders, such as preeclampsia (PE), are associated with increased apoptosis of trophoblast cells and impaired invasiveness which lead to inadequate remodeling of the uterine spiral arteries and shallow placentation [32,33,34]. In vitro studies with trophoblast cell lines have shown that like the PE placentas, the trophoblast cells exposed to increased oxidative stress exhibit altered invasion and migration properties, with changed expression of differentiation and invasion-associated molecular markers [35]. Also, it was shown that antioxidant molecules can reverse oxidative stress cascades, reduce cell damage and promote cell survival and invasiveness in trophoblast cells [36,37,38,39,40]. We have previously shown that OLE reduced cell damage in trophoblast cells caused by oxidative stress and improved antioxidant status of the cells [25]. In the same study, it was also shown that OLE is effective for reduction in the pro-inflammatory mediator iNOS, and cytokines IL-6 and TNF-α [25]. However, the impact of OLE treatment on the functional characteristics of trophoblast cells, such as adhesion, invasion and migration were not examined and the exact mechanism behind its action remained unresolved. As a next step towards the elucidation of the activity of OLE in trophoblast cells, the current study was designed to provide an answer whether OLE influences EVT invasion and adhesion and if its effects are mediated by COX-2 in conjunction with JNK signaling.

Cell proliferation and invasion are two key processes that are intrinsically coupled through matrix-adhesion friction but are being regulated by distinct pathways. These processes involve a series of well-controlled molecular players, including transmembrane receptors, adhesive complexes, cytoskeletal components, and the ECM [41]. To evaluate trophoblast cell movement several different in vitro assays such as the scratch assay, the invasion assay, and the proliferation assay are used collectively. These tests allow a thorough examination of potential mechanisms to identify which individual factors provide apparent changes in the cell migration [42]. In our study, results have shown that OLE does not alter (i) viability of HTR-8/SVneo cells in a range of concentrations 1–200 µM, and (ii) their proliferation rate after 24 h incubation with the two selected concentrations of 10 and 100 µM OLE. In studies on cancer cells, OLE showed in vitro anti-proliferative effect against human osteosarcoma cells at concentration higher that 200 µM [43]. In another study, OLE reduced testicular cancer cell line proliferation, promoted apoptosis and counteracted cell migration and motility at concentrations of 15–200 μM, while the exposure to same doses of OLE for 48 h did not affect HepG2 cell viability [44]. In a study with glioblastoma cell lines, OLE inhibited the viability, induced the apoptosis of U251 and A172 cells in vitro and showed inhibitory effects on migration and invasion [45]. It can be concluded that OLE has differential effects on cell proliferation and survival in normal vs. cancer cell lines and that its effects could be cell-type specific. 

Trophoblast cell invasion of the maternal decidua is a multistep process, similar to the invasion of a local tissue by malignant tumor cells. Although trophoblast cells inherently behave like metastatic cells in terms of spreading, their invasion is a tightly regulated process. It involves the attachment of trophoblast cells to components of the ECM, degradation of the matrix-by-matrix metalloproteinases (MMPs) and migration of trophoblast cells through the ECM [46]. The first step in this process is the attachment of the cells and their interaction with the network of secreted proteins and glycoproteins that form ECM. The ECM acts as ‘ground substance’ and it plays a vital role in trophoblast cellular adhesion. A functional adhesion test on different substrates (collagen and Matrigel) that imitate the properties of ECM was used to evaluate if OLE influences the adhesion of trophoblast cells after 24 h treatment. Our results have shown that OLE does not compromise the adhesion of HTR-8/SVneo cells on mentioned substrates. Further, we evaluated if OLE could alter the repertoire of molecules that mediate trophoblast adhesion and invasion. Namely, trophoblast cells interact with ECM components via specific cell-surface receptors called integrins, a class of transmembrane glycoproteins composed of α and β subunits, and the ligand-binding site comprised of parts of both chains [46]. Different integrin subunits combinations express distinct substrate preferences [47]. Trophoblast cells start to change their adhesive properties and integrin subunits repertoire on the surface as they acquire invasive phenotype. As they leave the basement membrane and acquire invasive phenotype, trophoblast cells increase the expression of α5β1 integrin, a fibronectin receptor. Further, as they progress within the uterine wall, they start to produce α1β1 integrin, a receptor for laminin and type IV collagen [47]. Our results have shown that OLE treatment at a lower concentration of 10 µM induced a significant increase in the mRNA expression of all examined integrin subunits (*ITGA1*, *ITGA5* and *ITGB1*), while higher OLE concentration of 100 µM did not change the mRNA expression of the mentioned subunits. The most pronounced change, a two-fold increase in mRNA expression, was observed for *ITGA1* after OLE treatment with 10 µM. Next, the change of protein expression of integrin α1 and β1 was also confirmed following the incubation with OLE at 10 µM. Together, these results indicate that the lower-dose treatment with OLE could influence invasive properties of trophoblast cells through stimulating expression of integrin subunits which mediate this process. So far, the influence of flavonoids and secoiridoids as natural integrin antagonists has been shown in cancer cells, where they effectively modulated a wide range of integrins, thereby inhibiting the key cancer hallmarks (adhesion, migration, proliferation, invasion, angiogenesis and metastasis) [48]. In contrast, within non-cancer cells such as trophoblast HTR-8/SVneo cells, the effects of flavonoids were associated with an increase in the invasive capacity [49]. Namely, the study by Ebegboni et al. [49] showed that flavonoids quercetin and hesperidin and their metabolites promoted invasion of HTR-8/SVneo cells, which is in accordance with our findings. Also, there is evidence supporting the enrollment of ROS in the reversible oxidation of integrins as redox-sensible proteins through a direct modification of the thiol groups, as proposed by Fiaschi et al. [50]. This oxidative modulation of the integrin molecules has a profound effect on cell spreading onto ECM, via a redox regulation of integrin signaling during cell adhesion. The same authors showed that ROS participate in actin reorganization during the dynamic process of ECM-induced cell spreading, and that gluthatione-depleted cells show impaired spread and loss of cytoskeleton organization [50]. Moreover, a study with ROS scavenger N-acetyl-l-cysteine showed that an antioxidant treatment can reverse ROS-induced effects, by upregulating β1 integrin activity and restoring the cell adhesion and spreading [51]. Considering all the aforementioned, redox regulation of protein function is the most probable explanation of the effects of OLE on integrin expression levels in trophoblast cells observed in our study. Our results showed that OLE can influence the invasive properties of HTR-8/SVneo cells by altering the expression of key effector molecules such as integrin subunits α1, α5 and β1 as well MMP-2 and MMP-9. These two MMPs are among the most significant proteinases produced by the implanting embryo as it invades into decidua [30]. Proteolysis of the basement membrane and ECM by MMPs forms the migration path for trophoblast. It is known that oxidative stress can influence the invasive and migratory behavior of trophoblast cells by modulating the matrix-degrading enzymes [35]. Both MMPs were stimulated in the treatments with the lower concentration of the OLE 10 µM in our study, indicating the OLE stimulates trophoblast cells towards pro-invasive behavior. In trophoblast cells, small levels of ROS promote the invasion while excessive ROS production exhausts cellular antioxidant defense systems, leading to the loss of invasive potential and reduced activities of MMP-2 and MMP-9, as shown by latest findings of Mukherjee et al. [52]. Our previous results showed that OLE can restore the antioxidant capacity of trophoblast cells exposed to H_2_O_2_ [25]. Thus, the ROS-mediated cellular response to OLE treatment could be facilitating the increased mRNA expression of *MMP2* and *MMP9* observed in this current study. 

Further, the study by Ebegboni et al. showed that flavonoids can modulate trophoblast cell invasion by reduction in oxidative stress levels through JNK/p38 mitogen-activated protein kinase (MAPK) signaling [49]. The MAPKs axis is a critical pathway for the propagation of the stress response, and the appearance of phosphorylated JNK is associated with stress conditions [53]. A study on animal models has shown that ROS-mediated apoptosis in trophoblasts was mediated predominantly by the activation of the JNK pathway [54]. Further, studies performed on villous explants in vitro showed that the activation of MAPK and the nuclear factor-κB pathways induced downstream consequences. These downstream effects included increased concentrations of TNF-α and IL-1β, increased expression of COX-2, and increased apoptosis [55]. Therefore, to assess whether the effects of OLE on HTR-8/SVneo cells are mediated by JNK-dependent signaling and COX-2, we analyzed the protein expression levels of the parameters in this signaling pathway. Treatment of trophoblast cells with OLE in our study showed inhibitory effects on both pJNK and COX-2 expression, indicating stress-reducing effects of OLE. It suppressed JNK activation in a concentration-dependent manner, while COX-2 was slightly reduced by both OLE concentrations to the same extent. Decrease in COX-2 expression is known to stimulate cell invasion in trophoblast cells [28]. Further, other authors observed increased expression and activity of COX-2 in the trophoblast cells and neutrophils from PE women [56,57]. Also, Adu-Gyamfi et al. showed an association between inhibited trophoblast invasion, decreased MMP-2 and MMP-9 expression and increased COX-2 expression [58]. Collectively, our results are in accordance with these findings and indicate that OLE treatment modulates the COX-2 and JNK-pathways, resulting in stimulated expression of trophoblast invasion markers. Further, analyzing the results of the cell migration assay, it can be also concluded that 24 h OLE treatment stimulated cell migration at both concentrations. The lower concentration in OLE exhibited a more pronounced effect on cell movement, which is in line with the other results obtained in this study. Numerous literature data show inhibitory effects of OLE on tumor cell migration and invasion in the same range of concentrations that was used in the current study [44,59,60]. On the other hand, there are very scarce results in non-cancer cells where OLE showed the opposite, stimulatory effects [61,62]. In that regard, it was shown that hydroxytirozol, a metabolite of OLE, stimulated migration of vascular endothelial cells and human umbilical vein endothelial cells [63,64]. Additionally, in the study on mouse fibroblasts, the OLE-containing fraction exerted the highest cell migration rate among other fractions of olive leaf extracts [61]. The same study showed that treatment with concentrations of 10 μg/mL (which is equivalent to 18.5 µM OLE) provided wound closure completely, while high concentrations > 50 μg/mL OLE (equivalent to 92.5 µM OLE) showed inhibitory effects on cell migration, indicating a critical concentration for stimulatory effects of OLE on cell migration. This is in line with our results in trophoblast cells, where 10 µM concentration of OLE was more effective than 100 µM OLE in promoting cell migration and stimulating the expression of trophoblast invasion markers. Such a discrepancy might stem from an imbalance in redox signaling by 100 µM OLE. This is not unusual given that some powerful antioxidants, including polyphenols, can generate ROS by autoxidation and redox-cycling or exert other cell deleterious effects in high concentrations [65,66,67]. Thus, OLE can play a role of the double-edged sword in cells, acting either as a pro-oxidant or an antioxidant, depending on its concentration [67].

## 4. Materials and Methods

### 4.1. Cell Line

The HTR-8/SVneo immortalized human EVT cell line (obtained by the courtesy of Dr. Charles H. Graham, Queen’s, Kingston, Canada) was used for in vitro experiments performed in this study. This cell line was selected for its phenotypic similarities to primary human EVTs, including the invasive ability in vitro. Cells were cultured in 25 cm^2^ flasks in complete Roswell Park Memorial Institute (RPMI) 1640 medium (Gibco, Thermo Fisher Scientific, Waltham, MA, USA) supplemented with 10% fetal calf serum (FCS, Pan Biotech, Aidenbach, Germany) and 1% antibiotic/antimycotic solution (Capricorn GmbH, Düsseldorf, Germany). Cells were propagated at 37 °C in a humid atmosphere in an incubator with 5% CO_2_. 

### 4.2. Preparation of Oleuropein for Cell Treatment

A stock solution of OLE was made by resuspending powdered OLE (Cas No. 32619-42-4, Extrasynthese, Genay, France) in dimethylsulfoxide (DMSO) to achieve a concentration of 100 mM. The stock solution was aliquoted and stored at −20 °C. For experimental work, the stock solution was further diluted in complete RPMI medium to achieve final OLE concentrations 1 to 200 µM. Based on the previously published results [25], for testing further effects of OLE on trophoblast cells, a concentration of 100 µM was chosen as the highest concentration of OLE that did not affect cell viability, while 10 µM OLE was chosen for testing the effects of a low concentration on HTR-8/SVneo cells. 

### 4.3. Cell Viability Assay

Crystal violet assay was used to assess cell viability after the treatment with OLE in a range of concentrations (1–200 µM), as described previously [68]. The cells were harvested from flasks after reaching 70% confluence, by using 0.25% trypsin-EDTA solution (Capricorn Scientific, Ebsdorfergrund, Germany) and counted. Next, the cells were seeded in 96-well plates at the density of 2 × 10^4^ cells/well in 100 µL of the complete medium. After allowing the cells to adhere for 24 h at 37 °C in a humidified incubator with 5% CO_2_, the medium was removed, and the preparations of OLE or fresh control medium (0.1% DMSO in complete RPMI medium) were added to the cells at volume of 100 µL/well. The treatments were incubated with the cells at 37 °C for 24 h. Following the incubation period, the treatments were removed, and cells were rinsed with phosphate-buffered saline (PBS). Plates with cells were dried and subsequently fixed with ice-cold acetone-methanol (1:1). The staining was performed with 0.05% crystal violet dye in 25% methanol and the incorporated dye was dissolved with 0.1 M sodium citrate in 50% ethanol at 100 µL/well. Optical density was read using a microplate reader (BioTek ELx800, BioTek® Instruments, Inc.,Vermont, USA) at 570 nm wavelength and the results are presented as a percentage out of control values. Each experiment was performed in triplicate and repeated three times. 

### 4.4. Cell Proliferation Assay

To investigate the effects of 10 µM and 100 µM OLE on HTR-8/SVneo cell proliferation, a carboxyfluorescein succinimidyl ester (CFSE) dilution-based proliferation assay was used [69]. In brief, 2 × 10^5^ HTR-8/SVneo cells were first stained with 5 μM CFSE (Sigma-Aldrich Chemie GmbH, St. Louis, MO, USA) for 10 min at 37 °C in the dark. Reaction was stopped by adding 5 × the staining volume of ice-cold PBS supplemented with 5% FCS. Next, labeled cells were seeded in 24-well plates in complete culture medium and left to adhere for 24 h. The next day, 10 µM and/or 100 µM OLE were added to the wells and incubated for 48 h in a humidified atmosphere of 5% CO_2_ at 37 °C. Untreated cells, both unstained and stained with CFSE, served as controls. After the incubation period, cells were harvested, washed two times with cold PBS and 4 µL of propidium iodide (100 µg/mL) was added to discriminate dead cells. For analysis, 30,000–50,000 events per sample were acquired on BD LSR II flow cytometer (Becton Dickinson, Mountain View, CA, USA). Cells were analyzed using FlowJo software version 10 (TreeStar Inc., Ashland, OR, USA). 

### 4.5. Functional Test of Cell Adhesion In Vitro

Here, 96-well plates were coated with collagen type I or Matrigel (BD Biosciences, San Jose, CA, USA), by adding 100 μL to each well, at a concentration of 250 ng/well in RPMI 1640 medium and left for 1 h at room temperature for gelation to occur. Next, the wells were washed with PBS, and non-specific binding was blocked by adding 1% bovine serum albumin (BSA) in PBS for 1 h at 37 °C. Afterwards, the plate was washed with PBS, dried and left at 4 °C with a desiccant until further use.

HTR-8/SVneo cells were seeded in a 24-well plate at a density of 2.5 × 10^5^ cells per well. The next day the treatments with 10 and 100 µM OLE in the complete medium were added to the cells. At the end of the treatment, cells were detached from the wells by adding 0.25% trypsin-EDTA solution (Capricorn Scientific, Ebsdorfergrund, Germany) and counted. A previously prepared 96-well plate was taken and the adhesion test of HTR-8/SVneo cells on collagen, Matrigel and uncoated plastic was performed according to the protocol described in the literature [70]. Suspensions of HTR-8/SVneo-treated cells were plated in coated or uncoated wells by seeding 2.5 × 10^4^ cells per well and incubated for 2 h at 37 °C. After incubation, the wells were carefully washed with PBS, and the cells were fixed with 50 μL of chilled acetone-methanol (1:1) solution for 10 min. After that, the plates were dried completely and then stained by adding 50 μL of 0.05% Crystal violet dye, as described above. The experiment was performed in triplicate and repeated in three independent experiments.

### 4.6. Cell Migration Assay

The influence of OLE on cell migration of HTR-8/SVneo cells was evaluated after 24 h treatment. The analysis was performed according to described method, [71] as follows: HTR-8/SVneo cells were plated at the density of 5 × 10^5^ in 6-well plates and incubated at 37 °C in 5% CO_2_ until reaching confluence. Cells were then scratched by using a sterile pipette tip and rinsed with PBS twice to remove detached cells. Fresh medium (2 mL) without or with treatment with OLE at 10 and 100 µM was then added to the cells, and the pre-selected fields were photographed at 0 h and 24 h. The width of the denuded area was measured using an electronic grid, and the distances crossed by the cells were determined. The experiment was repeated four times in duplicate and the results are presented as percentage out of control values. 

### 4.7. Determination of Gene Expression Using the Quantitative Real-Time PCR Method

A quantitative real-time PCR (qPCR) method was used to examine relative gene expression after OLE treatment in HTR-8/SVneo cells. Total RNA was isolated from treated cells according to the manufacturer’s instructions using TRIzol reagent (Thermo Fisher Scientific, USA). First-strand complementary DNA (cDNA) was synthesized from 1 µg of total RNA, using 0.5 µg of Oligo(dT) 12–18 primers (Thermo Fisher Scientific, USA), 250 µM of each dNTP and 200 U of RevertAid reverse transcriptase (Thermo Fisher Scientific, USA). qPCR was performed using a 7500 Real-Time PCR apparatus (Applied Biosystems, Waltham, MA, USA). The reaction mixture (10 μL) consisted of 1 μL cDNA template, 5 μL 2 × SYBR Green PCR Master Mix (Applied Biosystems, USA) and specific primers at a final concentration of 0.5 μM. For each analyzed gene, for each sample from different treatments, reactions were prepared in duplicate. Amplification of PCR products was performed by selecting the temperature program: 95 °C for 10 min, 40 cycles of 15 s at 95 °C and 1 min at 60 °C. Expression levels of matrix metalloproteinases 2 and 9 (*MMP2*, *MMP9*), integrin subunits α1, α5 and β1 (*ITGA1*, *ITGA5*, *ITGB1*) were normalized against *GAPDH* used as a reference. Calculations were made using the comparative 2^−ΔΔCt^ method.
*MMP2* F: TGCGACCACAGCCAACTACG *MMP2* R: ACAGACGGAAGTTCTTGGTGTAGG*MMP9* F: TGACAGCGACAAGAAGTG *MMP9* R: CAGTGAAGCGGTACATAGG*ITGA1* F: GGTTCCTACTTTGGCAGTATT *ITGA1* R: AACCTTGTCTGATTGAGAGCA*ITGA5* F: GGCAGCTATGGCGTCCCACTGTGG *ITGA5* R: GGCATCAGAGGTGGCTGGAGGCTT*ITGB1* F: GTGGTTGCTGGAATTGTTCTTATT *ITGB1* R: TTTTCCCTCATACTTCGGATTGAC*GAPDH* F: GAAGGTGAAGGTCGGAGT *GAPDH* R: GAAGATGGTGATGGGATTTC

### 4.8. Western Blot Protein Analysis

In order to examine the effect of OLE on protein expression, HTR-8/SVneo cells were seeded in 6-well plates (at a density of 5 × 10^5^ cells per well) and allowed to adhere overnight in complete RPMI medium. The next day, the medium was changed and treatments with 10 and 100 µM OLE were added to the complete medium and allowed to act on the cells for 24 h in an incubator at 37 °C, 5% CO_2_. Cells were washed 3 times with chilled PBS and cell lysates were prepared by adding cold RIPA buffer (Sigma Aldrich, St. Louis, MO, USA). Cells with RIPA buffer were left on a shaker at 4 °C for 30 min, after which lysates were collected and centrifuged at 4 °C for 20 min at 12,000 rpm to remove insoluble products. The supernatant was collected, poured into tubes and stored at −80 °C. Protein concentration in cell lysates was determined using a biquinonic acid assay kit (BCA kit, Thermo Scientific, USA), according to the manufacturer’s instructions. The absorbance at 540 nm wavelength was determined on a plate reader (Wallac 1420 multilabel counter Victor 3V, PerkinElmer, Waltham, MA, USA). The protein concentration was determined based on the standard curve, in the concentration range of 25–2000 μg protein/mL. The protein expression was determined by the immunochemical Western blot method according to the described method. Cell lysate samples in RIPA buffer were mixed with sample buffer (250 mM Tris-HCl pH 6.8, 2-mercaptoethanol, 30% glycerol, 0.1% bromophenol blue) and denaturation was performed by boiling for 5 min at 95 °C. Then, equal amounts of protein were loaded onto the gel. Electrophoresis was performed on a 12% polyacrylamide gel with sodium dodecyl sulfate (sodium dodecyl sulfate polyacrylamide gel electrophoresis, SDS-PAGE), and the levels of protein expression were determined by Western blot analysis using primary mouse monoclonal antibody Phospho-JNK1/JNK2 (1:2000; Thermo Fisher Scientific, USA), primary rabbit polyclonal antibody JNK1/JNK2 (1:2000; Invitrogen, Waltham, MA, USA) and secondary antibodies (1:2000, Anti-rabbit IgG, HRP-linked Antibody 7074P2, Cell Signaling Technology, Danvers, MA, USA, and 1:2000, Anti-mouse IgG, HRP-linked Antibody 7076S, Cell Signaling Technology, Danvers, MA, USA). Ponceau S staining was used as loading control. The protein bands were detected using enhanced chemiluminescence (ECL, SERVA Electrophoresis GE GmbH, Heidelberg, Germany). The Image-Master TotalLab v2.01 program (Amersham Biosciences, Inc., Piscataway, NJ, USA) was used to analyze intensity of bands, and the values of phospho-JNK1/JNK2 (pJNK) were normalized to the corresponding total JNK1/JNK2 values. Results were presented as percent of the value obtained for control cells.

### 4.9. Determination of Protein Expression Using the CELISA (CELL-BASED ELISA) Method

Cell-based ELISA was performed according to described method [72]. Namely, HTR-8/SVneo cells were seeded in 96-well plates at a density of 2 × 10^5^ cells per well and grown for 24 h at 37 °C and 5% CO_2_. The following day, medium was replaced with treatments containing 10 and 100 μM OLE in complete medium and incubated for 24 h with the cells. At the end of the treatment, the medium was removed, and the cells were washed twice with PBS, after which the plate was dried. After drying, the cells were fixed with ice-cold acetone-methanol (1:1) for 10 min. Afterwards, endogenous peroxidases were blocked by adding 0.3% H_2_O_2_, 200 μL per well for 30 min in the dark. Next, the wells were washed with PBS and blocked with the addition of 1% BSA in PBS for 30 min at 37 °C. After blocking, 50 μL of each primary antibody for α1 integrin subunit (MAB5676, source: mouse, 1:400, R&D Systems, MN, USA) or β1 integrin subunit (AB1952, source: rabbit, 1:500, Millipore Sigma, Burlington, MA, USA) or COX-2 (PA5-27238, source: rabbit, 1:500, Invitrogen, Waltham, MA, USA) was added in PBS with 1% BSA to the wells and incubated overnight at 4 °C in a humidified chamber. The next day, after the plate was washed five times with PBS containing 0.5% Tween, a secondary antibody (1:2000, Anti-rabbit IgG, HRP-linked Antibody 7074P2, Cell Signaling Technology, Danvers, MA, USA; or 1:2000, Anti-mouse IgG, HRP-linked Antibody 7076S, Cell Signaling Technology, Danvers, MA, USA) in PBS with 1% BSA was added to the wells and incubation lasted 1 h. Following the incubation, the plate was washed five times with PBS and 50 μL of substrate was added to each well, and color development was monitored. When the color developed, 50 μL each of stop reagent was added and the plate was read at 450 nm wavelength on a plate reader (BioTek ELx800, USA).

### 4.10. Statistical Analysis

One-way analysis of variance (ANOVA) with Tukey post-hoc test was used to assess differences in treatments versus control, after data were tested for normality. All results are expressed as mean + standard error of the mean (mean + S.E.M). GraphPad Prism 6.0 (GraphPad Software, Inc., La Jolla, CA, USA) was used for statistical analysis, where *p* < 0.05 was considered significant.

## 5. Conclusions

Based on the observed results, it can be concluded that OLE alone did not compromise survival, proliferation and adhesion of HTR-8/SVneo cells under the described conditions in vitro, which proves its safety. The results confirmed that OLE can influence the invasive properties of HTR-8/SVneo cells by altering the mRNA expression of effector molecules such as integrin subunits α1, α5 and β1 and MMP-2 and -9. Further, OLE significantly alleviated the stress signaling pathway in HTR-8/SVneo cells by inhibiting pJNK and COX-2 expression. Finally, OLE significantly stimulated cell migration following the 24 h treatment in vitro. The obtained data represent the first insight into the influence of OLE on molecular processes crucial for human trophoblast invasive function and provide foundation for further studies of its role and potential application as a therapeutic antioxidant in some pathological pregnancies, including PE. Also, the presented results provide guidelines for further studies of other polyphenols that may be of importance in pregnancy.

## Data Availability

Data used in this study are available on request from the first author.
